# Novel *N*-phenyl-2-(aniline) benzamide hydrochloride salt development for colon cancer therapy

**DOI:** 10.3389/fphar.2024.1452904

**Published:** 2024-10-30

**Authors:** Yan Peng, Ying Peng, Wei Zhang, Siyi Zhang, Huiqian Peng, Zhen Li, Bo Li, Linyi Liu, Linsheng Zhuo, Zhen Wang, Junbo Wu, Weifan Jiang

**Affiliations:** ^1^ School of Pharmaceutical Science, Hengyang Medical School, University of South China, Hengyang, Hunan, China; ^2^ Department of Anus and Intestine Surgery, Affiliated Hengyang Hospital of Hunan Normal University and Hengyang Central Hospital, Hengyang, Hunan, China; ^3^ The First Affiliated Hospital, MOE Key Lab of Rare Pediatric Diseases, Hengyang Medical School, University of South China, Hengyang, Hunan, China; ^4^ National Health Commission Key Laboratory of Birth Defect Research and Prevention Hunan Provincial Maternal and Child Healthcare Hospital, Changsha, Hunan, China; ^5^ Qinghai Provincial Key Laboratory of Tibetan Medicine Research, Northwest Institute of Plateau Biology, Chinese Academy of Sciences, Xining, Qinghai, China

**Keywords:** hydrochloride, water solubility, pharmacokinetic properties, Topo I and COX-2 dual inhibitor, anti-colon cancer activity

## Abstract

**Introduction:**

*N*-phenyl-2-(aniline) analog **N53** is a previously discovered dual inhibitor of Topo I and COX-2, which exhibited significant anti-colon cancer activity *in vitro*, but the poor solubility and moderate anti-cancer activity *in vivo* hindered its further development.

**Methods:**

To rectify the suboptimal drug properties of **N53**, a series of salt forms were developed and further evaluated through *in vivo* and *in vitro* experiments.

**Results:**

The hydrochloride (**N53·HCl**) has a well-characterized crystal structure and its solubility reached 540.1 μg/mL, which is nearly 1,700 times higher than that of **N53** (0.32 μg/mL). Increasing the **N53** solubility consistently promotes its effective concentration, further enhancing the COX-2/Topo I inhibitory activity and the anti-tumor activity *in vitro* (IC_50_ values of 2.95 ± 0.08 μM for HT29 cells, 7.99 ± 0.85 μM for RKO cells, 10.94 ± 1.30 μM for HCT116 cells), as well as the anti-proliferative and pro-apoptotic activity. Meanwhile, its oral pharmacokinetic property *in vivo* is also improved. The elimination half-life (T1/2) is prolonged from 10.78 to 22.29 h, the maximum plasma concentration (C_max_) is increased 2-fold, and the area under the plasma drug concentration-time curve (AUC_0–∞_) is increased 3-fold. In colon cancer xenograft mouse models, the tumor inhibition rate of **N53·HCl** was 53.7%, superior to that of **N53** (34.7%). Moreover, the results of HE staining showed that **N53·HCl** had no obvious toxic effects and side effects on other organs, indicating that it was safe *in vivo*.

**Discussion:**

This study demonstrated that **N53·HCl** exhibits superior pharmacokinetic properties, anti-colon cancer efficacy, and safety, providing a promising drug candidate for colon cancer therapy.

## 1 Introduction

Colorectal cancer is the most common clinical tumor of the digestive system, the third most common malignancy, and the second most fatal, making it a major global public health problem (Sung et al., 2021). In the early stages of colon cancer, there are often no obvious symptoms, and most patients are already in an advanced stage of the cancer when they are found. Chemotherapy plays an important role in the treatment of advanced colon cancer. The main first-line therapeutic agents used clinically to treat colon cancer are 5-fluorouracil and oxaliplatin, but they have severe side effects due to their lack of selectivity. Therefore, there is a great need for the development of new chemotherapeutic agents for the treatment of colon cancer (Argilés et al., 2020).

Currently, the expression and activity of human Topoisomerase I (Topo Ⅰ) in tumor cells is often higher than that in normal tissues, especially in colon cancer (Mcleod et al., 1994). Inhibition of Topo Ⅰ enzyme activity could arrest the cell cycle and cause cell death, making Topo Ⅰ as a prime target for cancer therapy. Irinotecan is a potent Topo I inhibitor derived from camptothecin and served as a key component of first- and second-line regimens for metastatic colon cancer, with limitations such as toxic side effects and acquired resistance (Cunningham et al., 1998; Vallböhmer et al., 2006). Cyclooxygenase-2 (COX-2) has been shown be overexpressed in colon cancer, contributing to tumor angiogenesis, invasion, and progression (Singh et al., 2006; Kaczkowski et al., 2016; Lombardi et al., 2022). It was verified that the chemotherapy regimen combining irinotecan and celecoxib can enhance anticancer activity, while this combined therapy involved drug-drug interaction which prevented their clinical application (Argiris et al., 2005; Javle et al., 2007). The development of pharmaceutical molecules with dual inhibition of Topo I and COX-2 could not only circumvent the potential risks of combined administration but also provide an alternative for colon cancer therapy. Our laboratory has designed and synthesized a series of *N*-phenyl-2-(aniline) benzamide analogs with the potential for both Topo I and COX-2 inhibitory activity. Among them, compound **NHWL05053(N53)** was able to block the cell cycle at the G1/G0 phase, inhibit cell proliferation, and induce apoptosis through the mitochondrial pathway and ROS burst. On the other hand, **N53** inhibited the aberrant activation of the NF-κB/IκB pathway which is a major regulator of colon cancer cell proliferation, apoptosis angiogenesis, inflammation, and metastasis (Xiaoling Hu et al., 2022). However, the poor solubility of **N53** has led to unsatisfactory pharmacokinetic properties and suboptimal therapeutic effects, making it undesirable druggability and difficult to further develop.

Drugs with poor water solubility are often accompanied by suboptimal and low oral bioavailability, which may account for their undesirable efficacy and unpredictable clinical response (Li et al., 2012; Williams et al., 2013). Thus, it is imperative to identify solubility enhancement strategies for those poorly soluble candidates. Currently, the solubility enhancement techniques mainly include cyclodextrin or maltodextrin complexation (Bénédicte Pradines, 2014; Ana et al., 2015; Ferreira et al., 2015; Chattah et al., 2017; Agustina et al., 2018), solid dispersion (Patricia et al., 2014), nanocrystals, salt formation (Yohei Kawabata et al., 2011), etc. From a practical perspective, salt formation has become the preferred method for improving the physicochemical properties of drug candidates in the early stages of drug development due to its convenience, feasibility, and effectiveness. In addition, the salification of the active pharmaceutical ingredient is of great significance in reducing the cost, shortening the time to market, and extending the patent protection period for industrialization. It is reported that among the 129 new small molecule chemical drugs approved by the FDA from 2015 to 2019, there are 61 (accounting for 48%) pharmaceutical salts (Bharate, 2021), such as the hydrochloride Pecetatinib and Fedratinib (Roskoski, 2020). Given these advantages, salt formation is a well-established and readily available method to improve the pharmacology of drug candidates.

**FIGURE 1:**
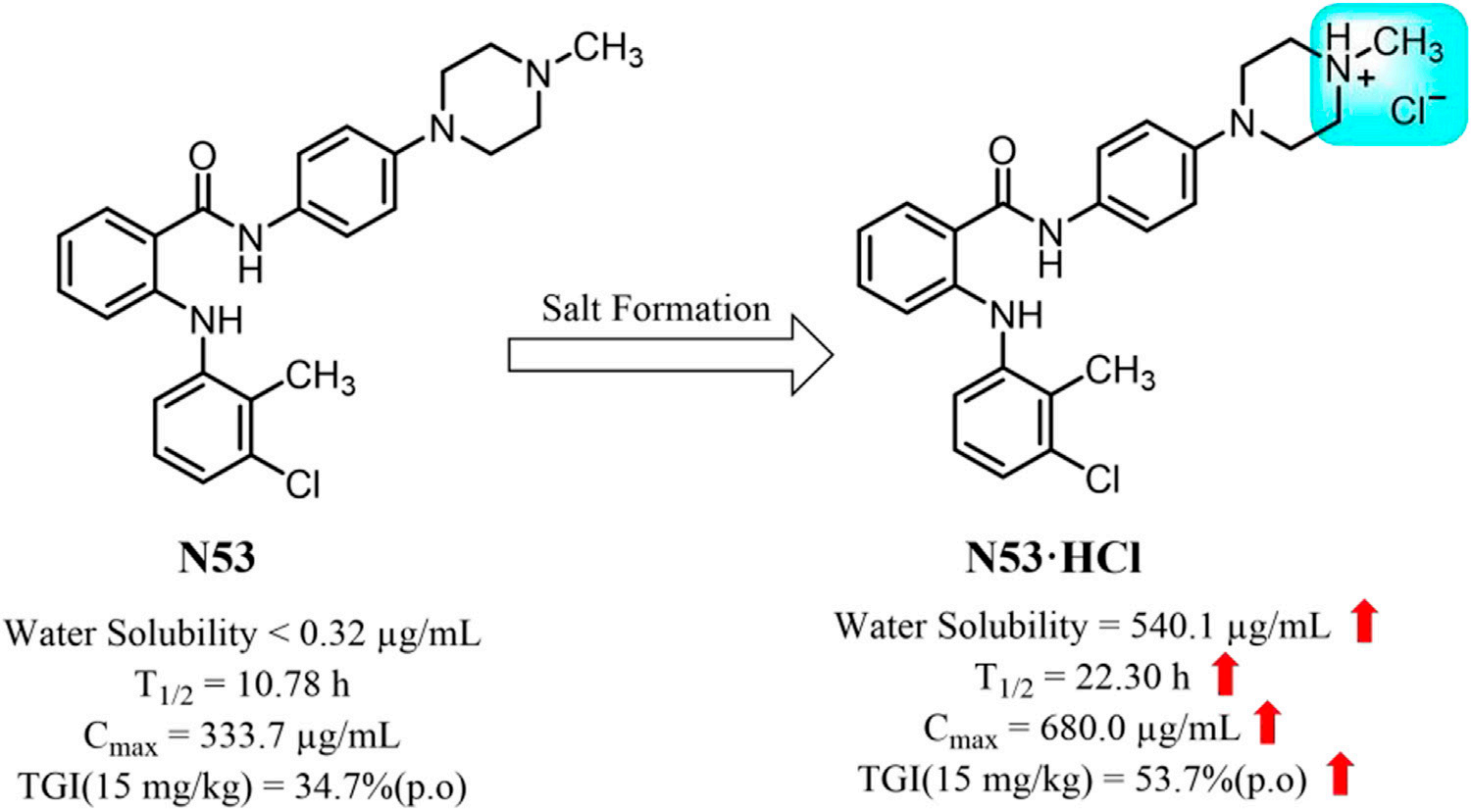
The optimal salt form of **N53** for colon cancer treatment.

**N53** is an *N*-phenyl-2-(aniline) benzamide derivative obtained by introducing a piperazine ring and exhibits stronger anti-colon cancer activity than other substituted derivatives (Xiaoling Hu et al., 2022). Along with this, the introduction of the piperazine ring makes it weakly alkaline, which provides a prerequisite for its salt formation. In this report, the different salt forms of **N53** were synthesized and screened, and it was found that **N53** hydrochloride (**N53·HCl**) is an ideal soluble salt with better oral pharmacokinetic properties and safety *in vivo*. Moreover, the proof-of-concept studies have shown that the salt form exerts promising anticancer efficacy in various colon tumor cells *in vitro*. Further studies in mouse xenograft models bearing human colon cancer confirmed this salt form’s *in vivo* anticancer activity. Collectively, **N53·HCl** may serve as a promising anti-colon cancer agent (Figure 1).

## 2 Materials and methods

### 2.1 Chemistry section

Unless otherwise noted, all reagents and solvents were commercially available and used without further purification. ^1^H NMR and ^13^C NMR spectra were obtained on either AVANCE500 spectrometers (Bruker Company, Germany) using DMSO-d6, CD_3_OD_3_ or CDCl_3_ as the solvent and TMS as the internal standard. Chemical shifts were given in ppm (d), and coupling constants (J) were reported in Hz. The purities of the target compounds were analyzed by HPLC (Agilent Technologies 1260 Infinity) using MeOH/H_2_O=90:10 as the mobile phase at a flow rate of 1 mL/min on a C18 column (Agilent 20RBA × SB-C18, 5 mm, 4.6 mm × 150 mm). All final compounds exhibited purities greater than 95%.

#### 2.1.1 General procedure for the preparation of 1-methyl-4-(4-nitrophenyl) piperazine (compound 2)

p-Fluoronitrobenzene (1 g, 7.1 mmol) and 1-methyl piperazine (708 mg, 7.1 mmol) were added to a 250 mL round bottom flask, then potassium carbonate (9.812 g, 71 mmol) was added. 30 mL of solvent dimethylsulfoxide was added and the reaction was carried out at 80°C for 12 h under argon protection. The dimethylsulfoxide was washed off by extraction with water and ethyl acetate, then dried with anhydrous magnesium sulfate, and 1.3246 g of yellow powder Intermediate 2 was obtained by silica gel column chromatography in 84% yield.

#### 2.1.2 General procedure for the preparation of 4-(4-methylpiperazin-1-yl) aniline (compound 3)

Intermediate **2** (1.3246 g, 6.0 mmol) was placed in a 100 mL aubergine flask and 27 mL of 2 mol/L dilute hydrochloric acid and 30 mL of methanol solvent were added. The reaction was heated to reflux at 60°C for 1 h under argon protection and then 5.7 g of zinc powder was added for 6 h. The solution was allowed to cool to room temperature and then brought to pH = 13 with 2 mmol/L sodium hydroxide and saturated sodium sulfate solution, filtered and extracted three times with ethyl acetate, the organic layers were combined, dried over anhydrous sodium sulfate and concentrated under reduced pressure to give 0.6782 g of white intermediate **3** in a calculated yield of 60%.

#### 2.1.3 General procedure for the preparation of compound N53

Tolmetic acid (0.926 g, 3.5 mmol) and 1-ethyl-(3-dimethyl aminopropyl) carbodiimide hydrochloride (0.847 g, 4.425 mmol) were added to a 100 mL round bottom flask with 30 mL dichloromethane as solvent and the reaction was carried out at room temperature under argon protection for 30 min, followed by the addition of Intermediate **3** (0.6782 g, 3.5 mmol), followed by 4-dimethylaminopyridine (0.108 g, 0.9 mmol) and triethylamine (1.1 g, 10.62 mmol) was added for 8–12 h. The organic phase was extracted three times with dichloromethane, combined, dried over anhydrous sodium sulfate, and concentrated under reduced pressure and column chromatography to give 0.9824 g of Intermediate 4 as a white solid in 64% calculated yield.

#### 2.1.4 General procedure for the preparation of compound N53·HCl

Preparation of the end product **N53·HCl**: 0.2 g of **N53** was added in 2 mL of methanol to dissolve it completely, adding oleic acid drop by drop, at which point a solid would precipitate, continuing to add acid until the solid no longer precipitated, and filtering and drying to obtain 0.18 g of the desired salt-shaped compound powder.

#### 2.1.5 Compound 2

Yellow solid (87% yield)^**. 1**^ **H NMR** (500 MHz, DMSO) δ 8.09–7.99 (m, 2H), 7.06–6.94 (m, 2H), 3.42 (t, *J* = 5.2 Hz, 4H), 2.40 (t, *J* = 5.2 Hz, 4H), 2.20 (s, 3H). ^**13**^ **C NMR** (126 MHz, DMSO) δ 154.7, 136.8, 125.7, 112.6, 54.1, 46.2, 45.6.

#### 2.1.6 Compound 3

Yellow solid (45% yield). ^**1**^ **H NMR** (500 MHz, DMSO) δ 6.76–6.63 (m, 2H), 6.48 (d, *J* = 8.6 Hz, 2H), 4.56 (s, 2H), 2.97–2.80 (m, 4H), 2.49–2.34 (m, 4H), 2.19 (s, 3H). ^**13**^ **C NMR** (126 MHz, DMSO) δ 142.4, 142.1, 117.8, 114.7, 55.0, 50.2, 45.8.

#### 2.1.7 Compound N53

White solid (77% yield). ^**1**^ **H NMR** (500 MHz, CDCl_3_) δ 9.18 (s, 1H), 7.77 (s, 1H), 7.55 (s, 1H), 7.47–7.41 (m, 2H), 7.30–7.27 (m, 1H), 7.21 (dd, *J* = 7.8, 1.4 Hz, 1H), 7.12 (dd, *J* = 8.0, 1.4 Hz, 1H), 7.07 (t, *J* = 7.9 Hz, 1H), 6.98 (d, *J* = 8.4 Hz, 1H), 6.96–6.92 (m, 2H), 6.82–6.77 (m, 1H), 3.19 (t, *J* = 5.0 Hz, 4H), 2.63–2.55 (m, 4H), 2.36 (s, 3H), 2.34 (s, 3H). ^**13**^ **C NMR** (126 MHz, CDCl_3_) δ 167.8, 148.9, 146.5, 141.2, 135.6, 132.7, 130.2, 129.8, 127.5, 126.8, 124.6, 122.5, 120.9, 118.1, 117.9, 116.7, 115.5, 55.2, 49.5, 46.3, 15.1. The purity was 95.926% by HPLC analysis.

#### 2.1.8 Compound N53·HCl

White solid (87% yield). ^**1**^ **H NMR** (500 MHz, DMSO) δ 10.97 (s, 1H), 10.28 (s, 1H), 9.27 (s, 1H), 7.80 (d, *J* = 7.8 Hz, 1H), 7.60 (d, *J* = 8.6 Hz, 2H), 7.36 (t, *J* = 7.8 Hz, 1H), 7.24 (d, *J* = 7.9 Hz, 1H), 7.18 (t, *J* = 7.9 Hz, 1H), 7.13 (d, *J* = 7.9 Hz, 1H), 7.01 (t, *J* = 9.0 Hz, 3H), 6.91 (t, *J* = 7.5 Hz, 1H), 3.77 (d, *J* = 12.5 Hz, 2H), 3.47 (d, *J* = 11.7 Hz, 2H), 3.12 (dq, *J* = 32.7, 11.8 Hz, 4H), 2.80 (d, *J* = 4.5 Hz, 3H), 2.26 (s, 3H). ^**13**^ **C NMR** (126 MHz, DMSO) δ 167.4, 146.1, 144.5, 141.5, 134.4, 132.2, 131.6, 129.4, 127.5, 127.4, 123.3, 122.1, 119.5, 118.9, 118.4, 116.3, 115.3, 52.1, 45.9, 41.9, 14.5. The purity was 99.200% by HPLC analysis.

#### 2.1.9 Preparation and detection of single crystals

**N53·HCl** was dissolved in dichloromethane solvent and stirred at room temperature. During the stirring process, acetonitrile solution was slowly added to make the mixed solvent reach the critical state of solid precipitation but quickly dissolved. The resulting solutions were then filtered through 0.45 µm nylon filters into clean vials and placed on a refrigerator. After standing for 3–10 days, small transparent crystals precipitated. A suitable crystal (0.01 mm × 0.02 mm × 0.03 mm) was selected and mounted on a Bruker D8 Venture diffractometer with MoKα radiation (λ = 0.71073 Å) (or CuKα radiation (λ = 1.54178 Å) or CaKα radiation (λ = 1.34139 Å) for cell determination and subsequent data collection at 170 K. Using Olex2, the structure was solved with the ShelXT structure solution program using Intrinsic Phasing and refined with the ShelXL refinement package using Least Squares minimisation. CCDC 2179821 contains the supplementary crystallographic data for this paper. These data can be obtained free of charge via http://www.ccdc.cam.ac.uk/conts/retrieving.html (or from the Cambridge Crystallographic Data Centre, 12, Union Road, Cambridge CB2 1EZ, United Kingdom; fax: +44 1223 336033).

**N53·HCl** Crystal Data for C27H31Cl2N5O (*M* = 512.47 g/mol): monoclinic, space group P21/c (no. 14), *a* = 21.9970 (9) Å, *b* = 8.2672 (3) Å, *c* = 14.4907 (6) Å, *β* = 95.730 (2)°, *V* = 2,622.02 (18) Å3, *Z* = 4, *T* = 170.0 K, μ(MoKα) = 0.277 mm^−1^, *Dcalc* = 1.298 g/cm^3^, 42,212 reflections measured (5.268° ≤ 2Θ ≤ 54.228°), 5,805 unique (*R*_int_ = 0.0590, Rsigma = 0.0351) which were used in all calculations. The final *R*1 was 0.0382 [I > 2σ(I)] and *wR*2 was 0.1029 (all data).

#### 2.1.10 Solubility assay

The thermodynamic solubility of **N53·HCl** in water was determined by high-performance liquid chromatography (Waterage, Alliance HPLC E2695, United States). The HPLC mobile phases were water (0.1% formic acid) and methanol separated by gradient chromatography at 0.5 mL/min. The chromatographic conditions were followed. Mobile phase: methanol: H_2_O = 10:90; 15 min; wavelength: 254 nm; temperature: 25°C. **N53** and **N53·HCl** were dissolved in methanol to form a gradient concentration standard solution (0.32, 1.6, 8, 40, 200 and 1,000 μg/mL). The standard solutions were scanned at 254 nm and the absorption area was recorded against the concentration curve. Saturated solutions of the compounds were prepared by magnetic stirring at 37°C for 4 h in deionized water in small volumes and the solid compounds were removed using Millipore 0.45 μm filters. The saturated solution was then scanned at 254 nm. The thermodynamic solubility in water was calculated from the absorption area and curve.

### 2.2 Biology section

#### 2.2.1 MTT assay

The anti-proliferation activities of synthesized compounds to colon cancer cell lines were measured by the MTT (3-[4,5-dimethyl-2-thiazolyl]-2,5-diphenyl-2H-tet razolium bromide) assay. First, the cell suspension was prepared by trypsin and the corresponding medium, cells were diluted to 5 × 10^5^ cells/mL, and 96-well plates were added 200 μL per well. The plates were maintained at 37°C in a humidified atmosphere containing 5% CO_2_ overnight. Then compounds at gradient concentrations were added to cells in the logarithmic phase to make the final concentration at 0.5, 1, 2, 4, 8, 16, and 32 μM. The cells were treated with tested compounds for 72 h, and the growth curve assay was tested in 24 h. When the time was up, 20 μL of MTT solution (5 mg/mL in PBS) was added into each well to incubate for 4 h at 37°C. Then the supernatant liquid was removed, 150 μL DMSO was added into every well to dissolve formazan crystals and read the absorbance at 570 nm by Spark microplate spectrophotometer (Tecan, Swiss). The IC_50_ values were analyzed by IBM SPASS Statistics 25.0 software. The data are all derived from three parallel experiments (n = 3) and expressed as mean ± SD.

#### 2.2.2 Topo I-mediated DNA relaxation assay

In brief, 2 μL Topo I Assay Buffer, 2 μL 0.1% BSA solution, 1 μL 1 U/μL Topo I solution, 0.2 μL test compound solution, and 0.5 μL DNA solution (0.25 μg) were added orderly with the corresponding volume of ultrapure water to make the above system volume 20 μL (Takara Biotechnology, Dalian, China). The above system was incubated at 37°C, the incubation continued for 45 min, and then 10 μL of a mixture of equal phenol and chloroform organic solvent was added for extraction to terminate the reaction. The aqueous phase was separated by centrifugation, the corresponding volume of 6 × DNA loading buffer was added, and then 0.8% agarose gel and 1 × TAE electrophoresis solution were used for electrophoresis at 110 V for 1 h. After electrophoresis, the gel was placed in a 0.5 μg/mL of ethidium bromide (EB) solution for 30 min, then recorded and photographed at 302 nm wavelength.

#### 2.2.3 COX-2 inhibition assay

The COX-2 inhibitory activity of all the synthesized compounds was measured by the Human COX-2 Inhibitor Screening Kit (Beyotime, catalog no. S0168), and the specific steps were followed strictly according to the instructions. The lead compound **N53** was also tested for reference purposes. The absorbance was measured at an excitation wavelength of 560 nm and an emission wavelength of 590 nm in a microplate reader. Each experimental condition was performed in triplicate and averaged.

#### 2.2.4 Plane colony assay

In 6-well plates, HT29 cells and RKO cells (1 × 10^3^ cells/well) were inoculated and maintained overnight. Then treated with **N53** and **N53·HCl** at different concentrations for 24 h and with normal culture-medium for 7 days respectively. After that, removed supernatant and fixed by 4% paraformaldehyde for 15 min. Then fixed cells were stained with 1% crystal violet dye for 15 min and rinsed with water. Finally, colonies were counted according to rules that nonoverlapping groups of at least 50 cells. The data are all derived from three parallel experiments (n = 3) and expressed as mean ± SD.

#### 2.2.5 Cell apoptosis by flow cytometer

Flow cytometer and Annexin V-FITC/PI Apoptosis Detection Kit were used to assess the influence of **N53·HCl** on HT29 cells and RKO cell apoptosis. Briefly, colon cancer cells were inoculated into a 6-well plate overnight to make sure every well contained 5 × 10^5^ cells. Then cells were incubated with **N53**, **N53·HCl**, and 5-Fu at different concentrations for 24 h respectively. After collecting 2 × 10^5^–1 × 10^6^ cells and washing cells with pre-cold PBS twice (1,500 rpm, 4°C, 5 min), 100 μL 1 × Binding Buffer, 5 μL Annexin-V-FITC and 10 μL PI Staining Solution was added successively and the mixture was incubated in the darkness for 30 min. After diluting with 400 μL of 1 × Binding Buffer, cells were detected in an Analyzer flow cytometer.

#### 2.2.6 Measurement of intracellular ROS generation

In 6-well plates, RKO cells (10^5^ cells/well) were inoculated and maintained overnight, then treated with tested compounds for 24 h. After Trypsin enzymic digestion and washed with PBS (1,500 rpm, 4°C, 3 min), cells were dyed by 2′,7′-dihydro dichlorofluorescein diacetate (DCFH-DA) solution (Beyotime, diluted in serum-free medium) for 20 min. The lead compound **N53** was also tested for reference purposes. After that, cells were rinsed with serum-free medium, and diluted with 0.5 mL serum-free medium. Finally, the DCFH-DA labeled cells representing ROS were detected in the flow cytometer (Beckman, Germany). Statistical analyses were performed on FlowJo v10 software.

#### 2.2.7 Mitochondrial membrane potential assay

RKO and HT29 cells were seeded in six-well plates at a density of 6 × 10^4^ cells/well and incubated overnight. After 24 h of treatment with diverse concentrations of tested compounds, cells were collected and resuspended with 0.5 mL of cell culture medium, and then 0.5 mL of prepared JC-1 staining workup was added, and incubated at 37°C for half an hour. Next centrifugation was performed to remove the supernatant, and the labeled cells were washed twice with JC-1 staining buffer. Finally, cells were resuspended with 500 μL of JC-1 staining buffer and detected by flow cytometry (BD LSRF ortessa).

#### 2.2.8 Animal experiments

All animal studies were subject to the basics of the Institutional Animal Care and Use Committee (IACUC), and the protocol was approved by the Institutional Animal Ethics Committee of the University of South China.

#### 2.2.9 Pharmacokinetics study

Six 250–280 g male SD rats, which are 8-week-old, were randomly divided into 2 groups (n = 3). The dose selection is based on previously described (Xiaoling Hu et al., 2022). 100 mg/kg **N53** and **N53·HCl** dissolved in normal saline containing 2.5% polyoxyethylene castor oil and 2.5% DMSO was administrated by oral medications. The first group was given 100 mg/kg of **N53** by intragastric administration, and the second group was given 100 mg/kg of **N53·HCl** by intragastric administration. For intragastric administration, blood samples were collected from the orbits of the rats in heparin sodium tubes at 0, 5, 15, 30 min, 1, 2, 4, 6, 8, 12, and 24 h after dosing; The above blood samples were centrifuged (2,500 rpm, 6 min, 4°C) to plasma, and stored at −80°C to be analyzed by LC-MS/MS. Firstly, 5 times methanol was added to each serum sample and mixed well. After centrifugation at 1,600 rpm for 10 min at 4°C, the samples were filtered through a 0.22 μm filter for analysis. The HPLC analysis was carried out on an ODS-C18 column (4.6 mm × 50 mm, 3 μm). The HPLC mobile phases consisted of water (0.1% formic acid) (A) and 95% acetonitrile (including 0.1% formic acid) (B), separated by gradient chromatography at 0.4 mL/min. Chromatographic conditions: mobile phase: 0–0.5 min, acetonitrile: H_2_O = 10:90; 0.5–0.6 min acetonitrile: H_2_O = 100:0, 0.6–2.4 min, acetonitrile: H_2_O = 100:0, 2.4–2.5 min acetonitrile: H_2_O = 10:90; wavelength: 254 nm; column temperature: 25°C. The column eluate was directly introduced into ES-API. The MS/MS instrument was conducted using the electrospray ionization (ESI) source with a positive ion detection mode. The analytes (**N53·HCl**) and IS (Propranolol) were all detected by multiple reactions monitoring (MRM) scan mode of transitions from precursor ions to product ions at m/z 435.2→192.1 for **N53·HCl** (retention time: 1.54 min), m/z 260.0→116.0 for IS (retention time: 1.54 min). The optimized instrument parameters are as follows: declustering potentials (DP) at 51 V for **N53·HCl**, 78 V for IS; entrance potentials (EP) at 8V; collision energies (CE) at 34 V for **N53·HCl**, 25 V for IS; collision cell exit potentials (CXP) at 7 V; curtain gas (CUR) at a pressure of 10 psi; turbo spray voltage at 5,500 V. Data acquisition of LC-MS/MS was performed with AnalystTM 1.5 software package (Applied Biosystems, MA, United States). The pharmacokinetic parameters for **N53** and **N53·HCl** were calculated by using the non-compartment method (statistical moment) in DAS 2.0 software (Pharmacokinetics Institute of China).

#### 2.2.10 HT29 nude xenograft model

HT29 cells in the logarithmic growth phase were digested to a cell density of 8 × 10^6^/mL, and 100 μL suspension of HT29 was injected subcutaneously in the right axilla of nude mice. The dose selection is based on previously described (Xiaoling Hu et al., 2022). When the average tumor volume reached about 300 mm^3^, they were randomly divided into 3 groups: **N53·HCl** (15 mg/kg once a day, p.o.), **N53** (15 mg/kg once a day, p.o.), control group. 15 mg/kg **N53** and **N53·HCl** dissolved in normal saline containing 2.5% polyoxyethylene castor oil and 2.5% DMSO were administrated by oral medications, while the Control group was given an equal volume of blank solvent. The tumor volume was measured with a vernier caliper every other days and calculated the tumor volume according to Equation 1. After 13 days of treatment, the mice were sacrificed, the tumors were removed, the tumor weight was weighed, and the tumor growth inhibition rate was calculated. The calculation formula of tumor growth inhibition rate (TGI%) was calculated using Equation 2. The corresponding equations were as follows:

$$\text{Tumor volume }\left({\text{mm}}^{3}\right)=\left(W^{2}\times L\right)/2$$ (1)

$$\text{TGI}\%=\left[1-\left(T-T_{0}\right)\;/\;\left(C-C_{0}\right)\right]\times 100\%$$ (2)

In equations, W and L represent the width and length of the tumor respectively, T and C represent the average volume of the experiment group and control group at the end of the experiment. T_0_ and C_0_ represent the experiment group and the control group respectively.

### 2.3 Statistical analysis

All statistical analyses were performed with SPSS 22.0 (IBM, Chicago, IL, United States). Data are presented as the mean ± standard deviation (SD) and all experiments were performed at least twice. Student’s t-test (two-tailed) and a one-way ANOVA followed by a *post hoc* test were used when comparing two groups and multiple groups, respectively. A *P* < 0.05 was considered significant.

## 3 Results

### 3.1 Chemistry

#### 3.1.1 N53 salts synthesis

**SCHEME 1:**
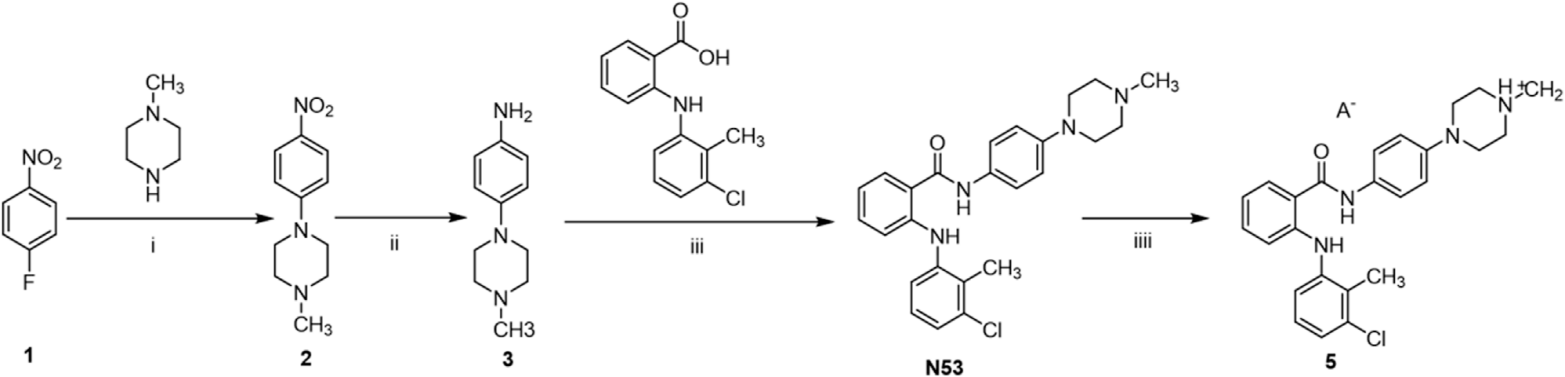
Synthesis of **N53·HCl**. Reagents and conditions: (i): K_2_CO_3_, DMSO, 80°C, rt (ii): Zn, HCl, 60°C, rt (iii): EDCl, DMAP, DCM, rt (iv): acid and solvent.

As shown in Scheme 1, the synthetic route of the target compound starts with p-fluoronitrobenzene, which undergoes a substitution reaction with 4-methylpiperazine in the presence of potassium carbonate, followed by a reduction with zinc powder to give the amino group, and then an acid-base condensation reaction with tromethamine to give the key intermediate compound **N53**. **N53** reacts with various organic acids as well as inorganic acids in solvents to produce the target **N53** in various salts. All compounds are characterized by NMR and X-ray, and the purity is guaranteed by HPLC to more than 95%, which meets the needs of biological experiments.

A series of organic acids and inorganic acids was used for **N53** salt formation. The organic acids we tested were L- (+)-tartaric, D- (+)-tartaric, citric, fumaric, oxalic, methyl-salicylic, maleic, L-melic, D-melic, benzenesulfonic, mesylate, succinic, and adipic. As for the inorganic acids, we used hydrochloric acid, hydrobromic acid, and sulfuric acid. However, even if modifying the reaction conditions, such as temperature, water control, acid reaction rate, etc., most acids, except *hydrochloric acid* and *hydrobromic acid*, could be successfully converted into salts to generate the corresponding organic salts. **N53·HCl** is low in hygroscopicity, relatively stable under various conditions, and has significantly higher solubility (540.1 μg/mL), which is nearly 1,700 times higher than that of **N53** (0.32 μg/mL). However, **N53-HBr** is highly hygroscopic and relatively unstable, transforming from a dry solid to a liquid state in just 4 h in air. After extensive consideration, the hydrochloride was selected.

#### 3.1.2 X-ray crystallography of N53·HCl

**FIGURE 2:**
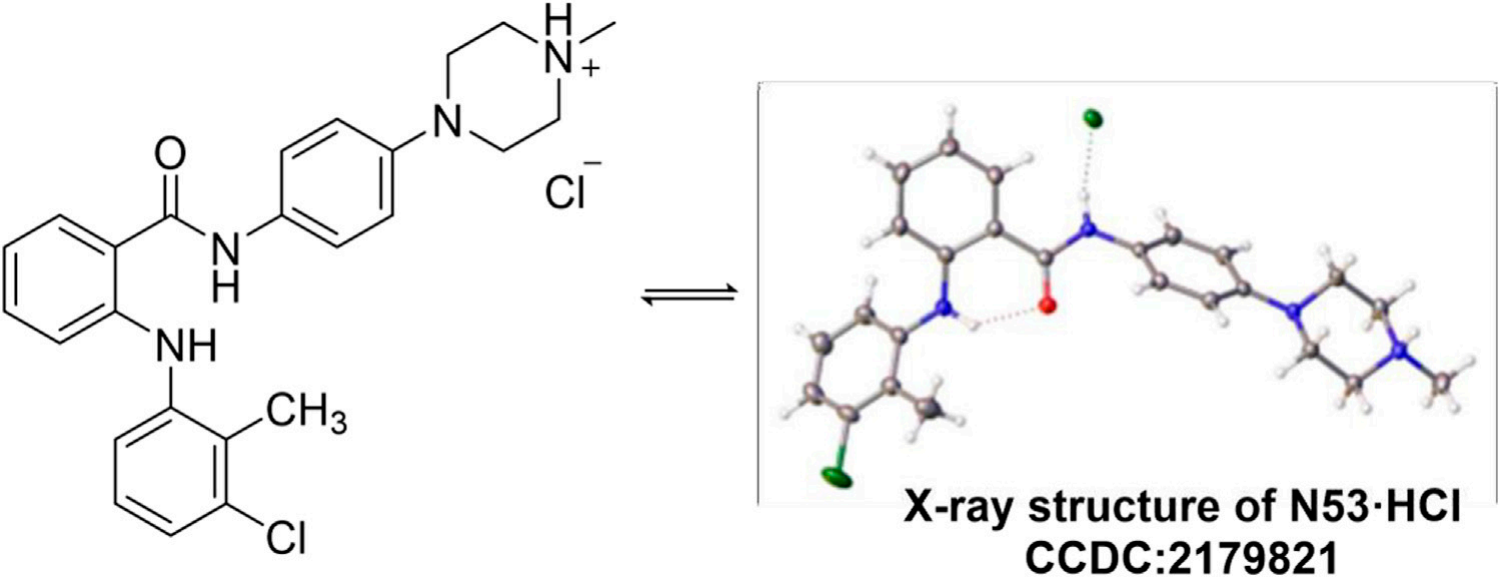
Single crystal diffraction of **N53·HCl**.

The single crystal of **N53·HCl** was obtained by slowly evaporating a mixture of dichloromethane and acetonitrile solution at ambient temperature. **N53·HCl** (0.01 mm × 0.02 mm × 0.03 mm) was selected and mounted on a Bruker D8 Venture diffractometer with MoKα radiation (λ = 0.71073 Å) (or CuKα radiation (λ = 1.54178 Å) or GaKα radiation (λ = 1.34139 Å) for cell determination and subsequent data collection at 170 K. Using Olex2, the structure was solved with the ShelXT structure solution program using Intrinsic Phasing and refined with the ShelXL refinement package using Least Squares minimisation. Analysis of the crystal structure of **N53·HCl** (Table 1) reveals that hydrogen atoms of hydrochloric acid attack N4 on the piperazine ring to form an N-H bond, and chlorine atoms form a hydrogen bond with N2-H on the amide bond at a distance of 2.436 Å. At the same time, N4 forms a tetrahedron-like spatial configuration with the surrounding three carbon atoms and hydrogen atoms. From the crystal structure, there is an intramolecular hydrogen bond between O1 and N1-H, and the distance is 2.015 Å. Crystallographic data for the structure reported in this paper have been deposited at the Cambridge Crystallographic Data Center and allocated with the deposition numbers: **CCDC 2179821** (Figure 2).

**TABLE 1:** Crystal data and structure refinement for **N53·HCl**.

Compd.	N53·HCl
Empirical formula	C_27_H_31_Cl_2_N_5_O
Formula weight	512.47
Temperature/K	170.0
Crystal system	monoclinic
Space group	P2_1_/c
a/Å	21.9970 (9)
b/Å	8.2672 (3)
c/Å	14.4907 (6)
α/°	90
β/°	95.730 (2)
γ/°	90
Volume/Å^3^	2,622.02 (18)
Z	4
ρ_calc_g/cm^3^	1.298
μ/mm^−1^	0.277
F (000)	1,080.0
Crystal size/mm^3^	0.43 × 0.29 × 0.08
Radiation	MoKα (λ = 0.71073)
2Θ range for data collection/^°^	5.268 to 54.228
Index ranges	−28 ≤ h ≤ 28, −10 ≤ k ≤ 9, −18 ≤ l ≤ 18
Reflections collected	42,212
Independent reflections	5,805 [R_int_ = 0.0590, R_sigma_ = 0.0351]
Data/restraints/parameters	5,805/0/319
Goodness-of-fit on F^2^	1.039
Final R indexes [I ≥ 2σ (I)]	R1 = 0.0382, wR_2_ = 0.0957
Final R indexes (all data)	R1 = 0.0507, wR_2_ = 0.1029
Largest diff. peak/hole/e Å^−3^	0.49/−0.41

### 3.2 The inhibitory effects of N53·HCl on COX-2 and DNA topoisomerase I

**FIGURE 3:**
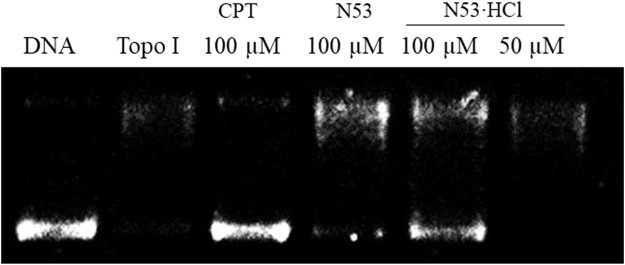
Compound inhibition of Topo I deconvolution of pBR322.

To further elucidate the effect of salt formation on the inhibitory activity against COX-2 and Topo I, the Topo I and COX-2 inhibitory activities of **N53·HCl** were evaluated. As shown in Table 2, **N53·HCl** showed a stronger ability to inhibit COX-2 activity than **N53** at 2 µM (Table 2). On the other hand, **N53·HCl** significantly inhibited Topo 1-mediated relaxation of supercoiled DNA at a concentration of 100 μM, while **N53** showed weak inhibitory activity at the same concentration (Figure 3; Table 2). It is speculated that salt formation could enable N53 to fully exert the inhibitory effects by increasing the solubility and effective concentration.

**TABLE 2:** Inhibition of COX-2 and Topo I activity.

Compd.	N53·HCl	N53
Inhibition rate (%) of COX-2 (2 μM)	32.5 ± 7.8	3.6 ± 1.1
Inhibition rate (%) of Topo I (100 μM)	55.1	11.7

### 3.3 N53·HCl inhibited the cell viabilities of various tumor cells

To verify that salt formation did not alter the anti-tumor activity of the prototype, the anti-proliferative activities of **N53·HCl** against human colon cancer cells (HCT116, RKO and HT29), hepatoma carcinoma cell (SK-Hep-1, HuH7, and HepG2) and prostate cancer cells (PC-3) were assayed. As shown in Table 3, the difference in anti-proliferative activity against various cancer cells between **N53·HCl** and **N53** was insignificant, indicating that hydrochlorination of **N53** does not affect its anti-proliferative activity *in vitro*.

**TABLE 3:** *In vitro* anti-proliferative activities of **N53·HCl**.

Compd.	IC_50_(µM) ± SD						
	SK-Hep-1	HuH7	HCT116	HepG2	PC-3	HT29	RKO
N53·HCl	8.18 ± 042	11.74 ± 0.75	10.94 ± 1.30	4.12 ± 0.39	13.74 ± 0.80	2.95 ± 0.08	7.99 ± 0.85
N53	5.66 ± 0.47	3.67 ± 0.38	3.89 ± 0.41	2.31 ± 0.09	11.26 ± 0.46	11.26 ± 0.46	6.29 ± 0.54

### 3.4 N53·HCl inhibited the proliferation of HT29 and RKO cells

**FIGURE 4:**
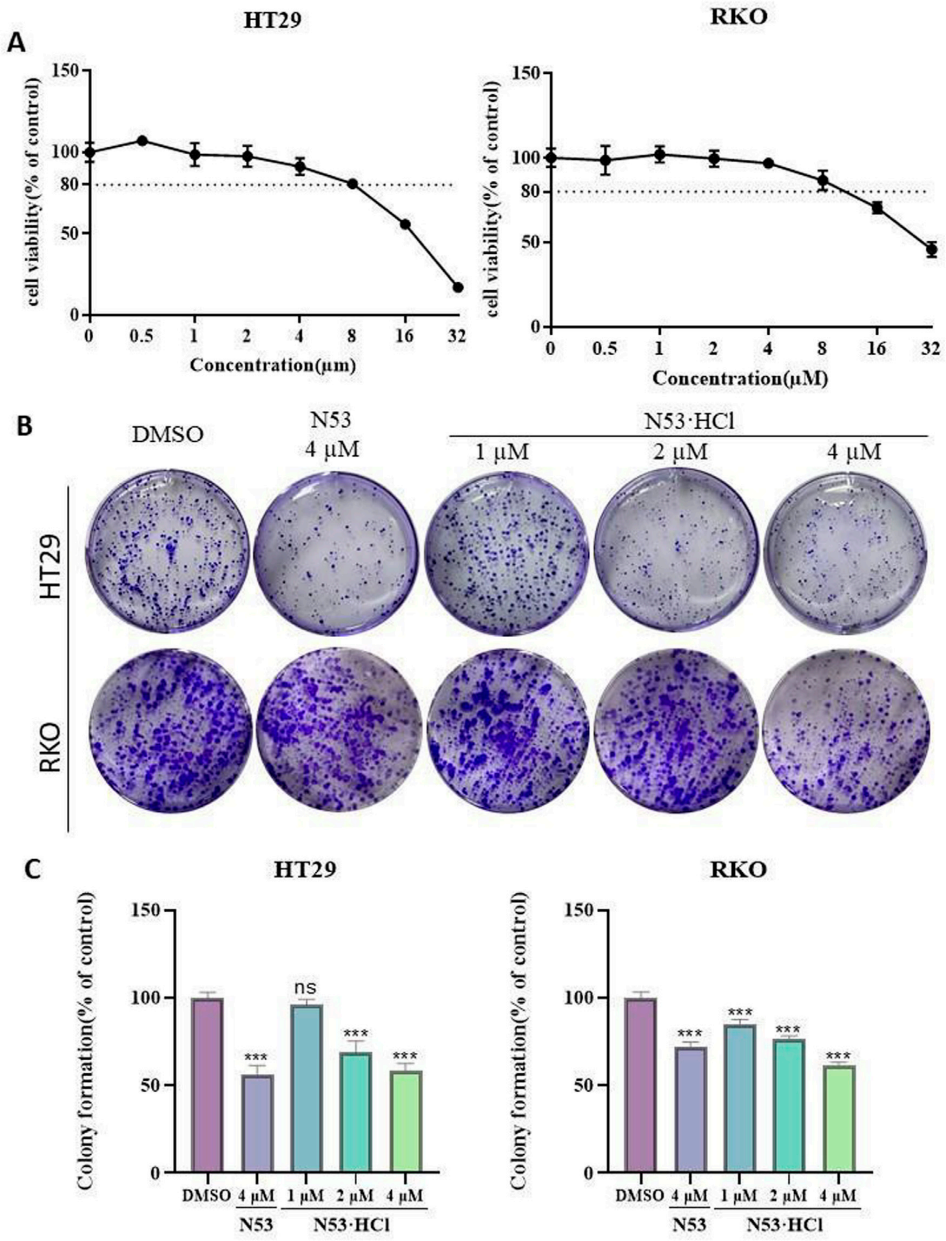
The effect of **N53·HCl** on the proliferation ability of HT29 and RKO. **(A)** The effect of **N53·HCl** on the cell viability of HT29 and RKO cells. HT29 and RKO were exposed to different concentrations of **N53·HCl** for 24 h, drawing the concentration-inhibition rate-time curve. **(B, C)** Plane colony assay to verify the inhibition of **N53·HCl** on cell proliferation. DMSO, different concentrations of **N53·HCl** were incubated with HT29 or RKO for 7 days to observe the formation of cell colonies. The data are all derived from three parallel experiments (n = 3). Compared with the DMSO group, *P < 0.05, **P < 0.01, ***P < 0.001.

To further determine the inhibitory effects of **N53·HCl** and **N53** on human colon cancer cells RKO and HT29 and to improve the basis for selecting drug concentrations for subsequent *in vitro* cellular assays, the MTT assay was used to determine the cell viability of different concentrations of **N53·HCl** and to plot the quantitative efficacy curves (Figure 4A). The results showed that the evaluation of *in vitro* experiments such as apoptosis was not affected by cytotoxic factors in the 24-hour range and concentration conditions. Therefore, HT29 and RKO cells were treated with 1, 2, and 4 µM **N53·HCl** for cell clone formation assay. The results showed that the number of cell clones formed in both cells decreased significantly and dose-dependently after treatment with this concentration gradient of **N53·HCl**, and a significant difference could be formed in HT29 cells with the control **N53** at the same concentration and conditions (Figure 4B). These results indicate that **N53·HCl** significantly enhanced the inhibition of colon cancer proliferation in HT29 cells compared with **N53**.

### 3.5 N53·HCl induced the apoptosis of HT29 and RKO cells

**FIGURE 5:**
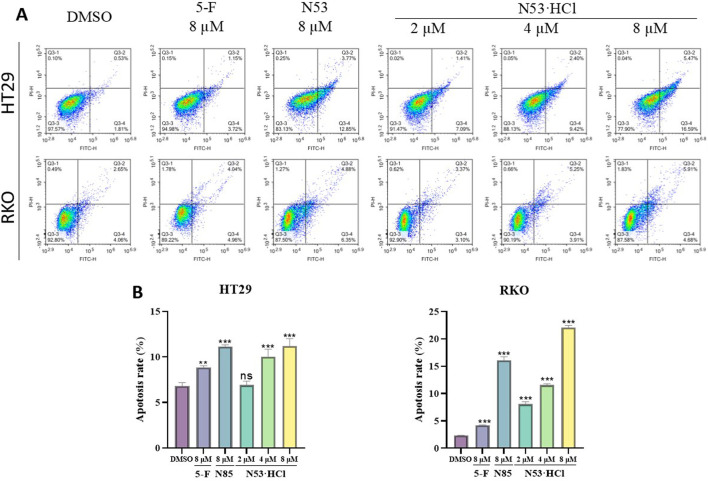
Different concentrations of **N53·HCl** induced apoptosis of HT29 and RKO cells after 24 h treatment. **(A, B)** Flow cytometry tested the types and proportions of apoptosis induced by **N53·HCl** in HT29 and RKO cells for 24 h. The data are all derived from three parallel experiments (n = 3). Compared with the DMSO group, *P < 0.05, **P < 0.01, ***P < 0.001.

After 24-hour incubation with concentrations of 2, 4, and 8 µM of **N53·HCl**, an increase in apoptotic cells and a change in cell morphology from spindle-shaped to roundish was observed microscopically with increasing the concentration of the compound. As shown in the graph, early apoptotic cells (region Q4) and late apoptotic cells (region Q3) increased with increasing concentration of the compound and induced apoptosis more effectively than **N53**, N53·HCl and its prototype drug N53 exhibited stronger activity in pro-apoptosis than the positive drug 5-fluorouracil (5-F). This indicates that **N53·HCl** can induce apoptosis in human colon cancer cells RKO and HT29 in a concentration-dependent manner and better than **N53**, as shown in Figure 5.

### 3.6 N53·HCl induced the ROS burst and mitochondrial dysfunction of HT29 and RKO cells

**FIGURE 6:**
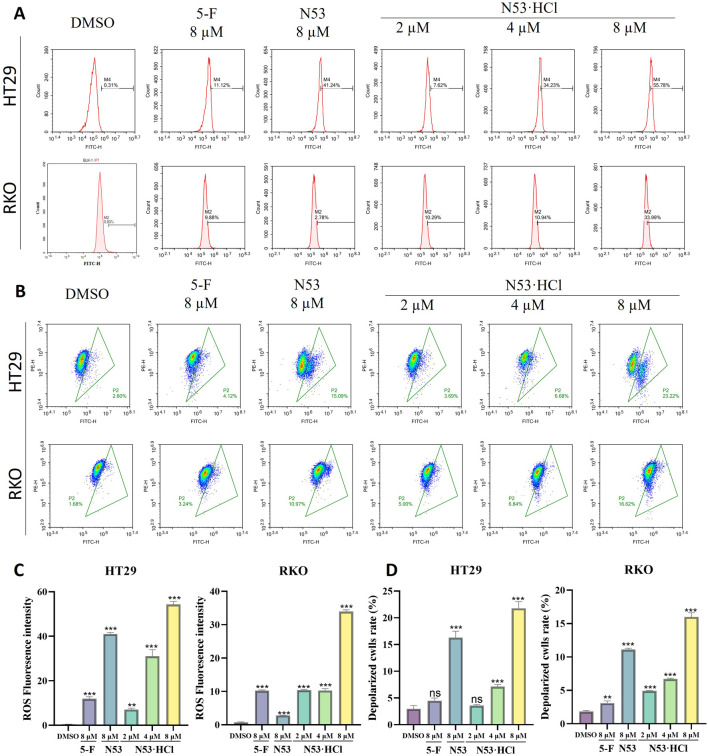
Effects of **N53·HCl** on mitochondria and ROS. **(A, C)** The number of DCFH2-DA-stained cells detected by flow cytometry and the statistical Graph (n = 3). **(B, D)** Populations of JC-1 stained cells detected by flow cytometry and the statistical Graph (n = 3). The data are all derived from three parallel experiments (n = 3). Compared with the DMSO group, *P < 0.05, **P < 0.01, ***P < 0.001.

Numerous studies have demonstrated a close relationship between mitochondrial dysfunction and apoptosis, with the decline of mitochondrial membrane potential (MMP) considered as one of the early events in the cascade of apoptosis. To determine whether **N53·HCl** induced apoptosis is related to the mitochondrial pathway, we detected MMP by JC-1 staining. As shown in Figures 6B, D, treatment with **N53·HCl** at high concentration induced the loss of MMP in HT29 and RKO cells, which was superior to **N53**. These results suggest that the apoptosis induced by **N53·HCl** is associated with the mitochondrial pathway.

Studies have shown that activation of ROS induces apoptosis in cancer cells. The above studies showed that **N53·HCl** induces apoptosis in human colon cancer cells (RKO and HT29), so reactive oxygen species assays were performed in this work. When **N53·HCl** was incubated at concentrations of 2, 4, and 8 µM for 24 h, flow cytometry revealed that **N53·HCl** was able to promote the production of ROS in a dose-dependent manner and was more effective than **N53** and the positive control drug 5-F (Figures 6A, C). This suggests that **N53·HCl** can promote the production of ROS to further induce apoptosis.

### 3.7 N53·HCl exhibited superior pharmacokinetic properties *in vivo*

**FIGURE 7:**
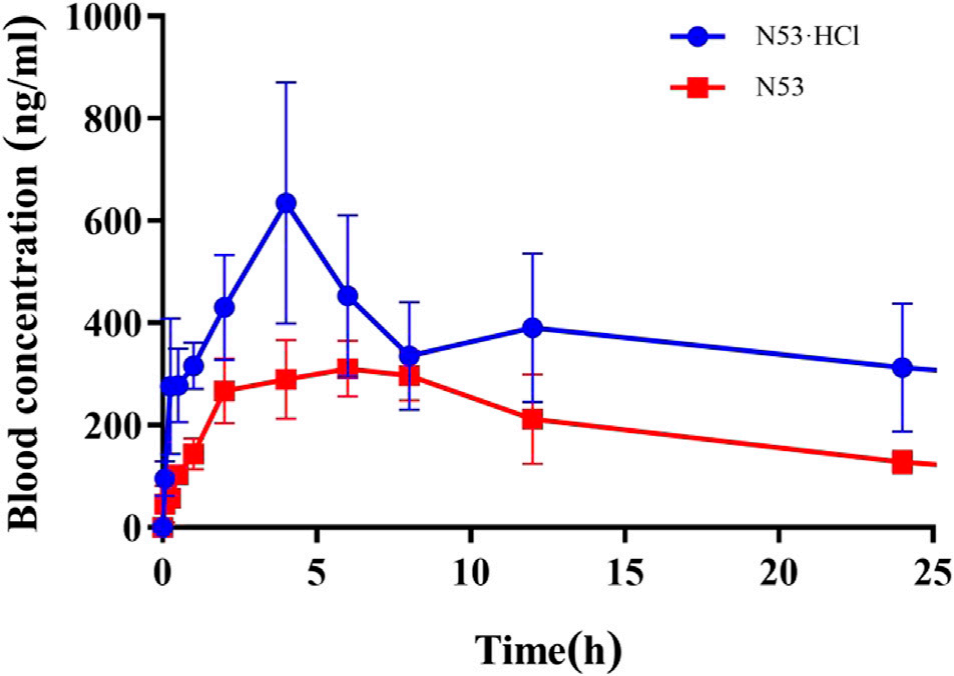
Blood concentration-time profiles of **N53·HCl** and **N53** in mice. Animals were treated with **N53** and **N53·HCl** with single doses of 100 mg/kg (p.o., n = 3) respectively.

*In vitro* cell assays show that **N53·HCl** has potent *in vitro* anti-tumor carcinoma activity, and in this work, the metabolic parameters of **N53·HCl** and **N53** are determined *in vivo*. As shown in Table 4; Figure 7, **N53·HCl** and **N53** were administered at a dose of 100 mg/kg by swallowing, and the plasma concentration of **N53·HCl** peaked at 680.0 μg/mL after 6.67 h, while **N53** peaked at 333 μg/mL with shorter T_max_ of 6.0 h. In addition, the half-life (T_1/2_) of **N53·HCl** was 22.3 h compared to 10.8 h for **N53**, indicating that the elimination rate of the drug *in vivo* was prolonged by hydrochloride formation. The area under the concentration-time curve (AUC_0–t_) for **N53·HCl** and **N53** were 21.2 and 7.2 mg/L × h, respectively, indicating that the drug exposure *in vivo* almost tripled, which greatly enhanced the absorption of the drug *in vivo*. Therefore, it can be seen that **N53** in salt to obtain **N53·HCl** can greatly improve the absorption *in vivo* and prolong the elimination time of the drug *in vivo*, therefore, further *in vivo* experiments are carried out in this study.

**TABLE 4:** *In vivo* pharmacokinetic properties of **N53** and **N53·HCl**.

Comp.	Dose (mg/kg)	Rout	ADME parameter ^a^					
			AUC0_(-∞)_ (mg/L h	T^1/2^ (h)	C_max_ (mg/mL)	T_max_ (h)	CLz (L/h/kg)	Vz/F (L/kg)
N53	100	p.o.	7.2 ± 0.7	10.8 ± 0.7	0.33 ± 0.04	6.0 ± 2.0	13.9 ± 1.3	217.1 ± 33.3
N53·HCl	100	p.o.	21.2 ± 8.0	22.3 ± 9.9	0.68 ± 0.18	6.7 ± 4.6	5.1 ± 1.6	149.7 ± 11.6

^a^ Pharmacokinetic parameters were calculated using DAS 2.0 software. All data were presented as the mean ± SD (n = 3).

### 3.8 N53·HCl oral dose was tolerated and safe *in vivo*

Acute toxicity studies were conducted for **N53·HCl** following OECD guidelines. Sixteen Kunming mice were purchased from SPF (Beijing) Biotechnology Co., Ltd. The mice were randomly divided into two groups (n = 8, 4 female and 4 male mice per group). The control group was treated with saline containing 10% DMSO, 10% Solutol HS -15, and the experimental group was treated with **N53·HCl** at a dose of 1,000 mg/kg (suspended in saline containing 10% DMSO and 10% Solutol HS -15).

**FIGURE 8:**
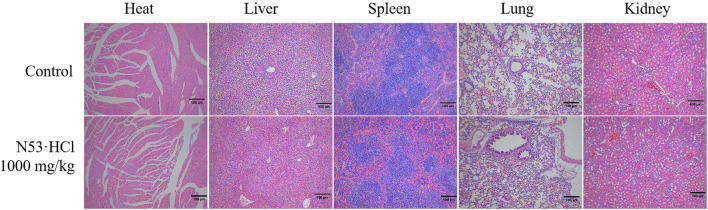
Histologic specimens of mice tissues (heart, liver, kidney, and spleen) after administration of **N53·HCl** were stained by hematoxylin and eosin (H&E) (Magnification ×100, scale bar = 100 µm).

All treatments were administered with a single dose by gavage. The mice were continuously monitored for 14 days and showed no significant changes in symptoms and body weight compared to the vehicle-treated control. After 14 days, the mice were sacrificed, and gross and histological examination of the major organs, including the heart, liver, kidney, and spleen, revealed no significant abnormalities (Figure 8). It can be seen that **N53·HCl** is a safe agent with an oral LD50 of more than 1,000 mg/kg in mice.

### 3.9 N53·HCl inhibited tumor growth in the HT29 xenograft model

**FIGURE 9:**
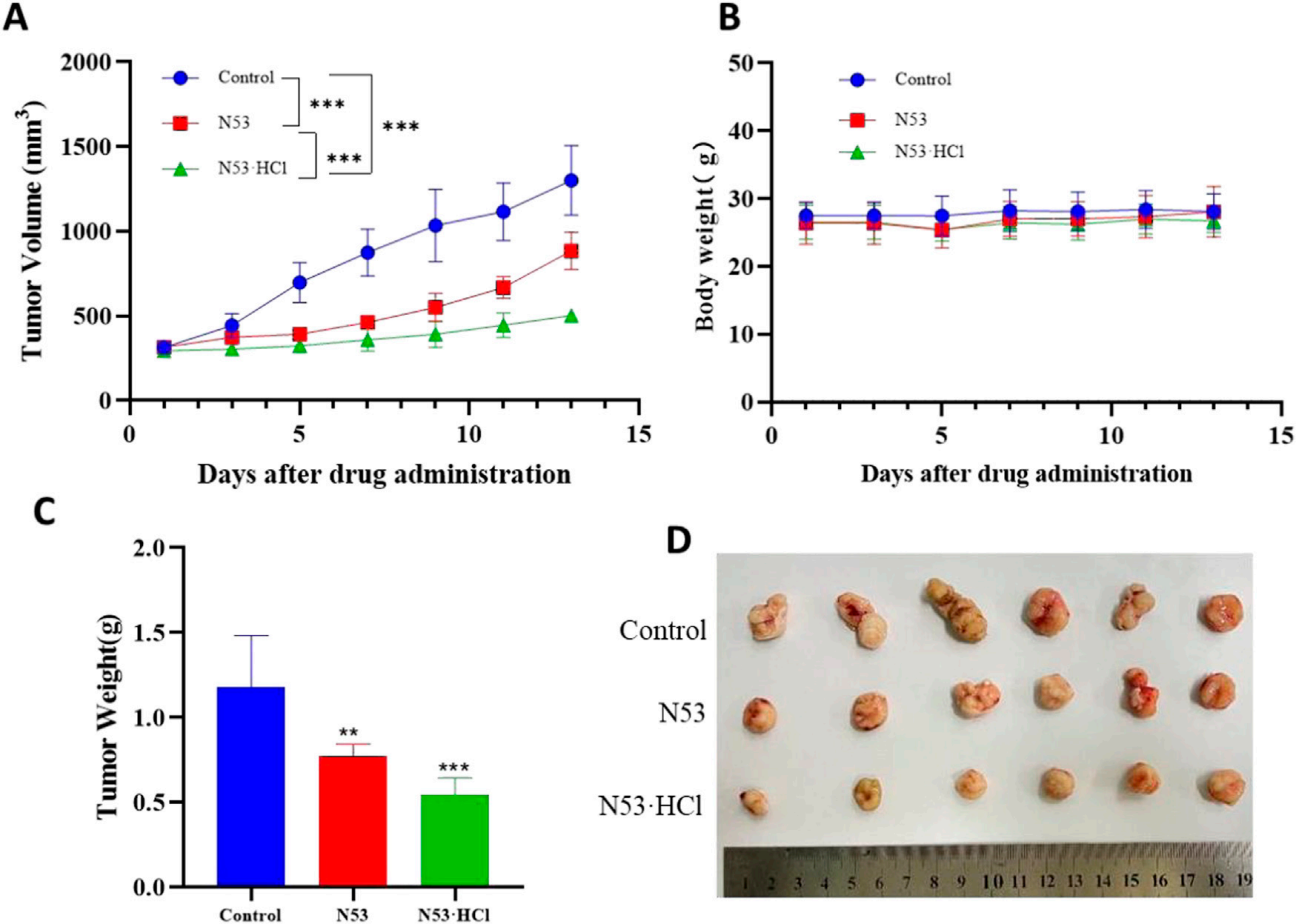
**N53·HCl** inhibited tumor growth. Male BALB/c nude mice were divided into three groups (n = 6) including the control, **N53**, and **N53·HCl**. Each group except the control group was given 15 mg/kg once a day for 13 days (p.o.). **(A)** Plot of tumor volumes over time, recorded every other days, plotted as mean ± SEM. **(B)** Diagram of mice weight over time, shown as mean ± SD. **(C, D)** Weight and size of tumors on the last day (day 13).

Based on pharmacokinetic and acute toxicity data and previous studies(Xiaoling Hu et al., 2022), a dosing interval of once daily was established and a dose of 15 mg/kg was administered, which was less than one-tenth of the acute toxicity dose, with **N53** selected as the control drug. After 13 days of administration, the mice had their tumors removed, and the results of the experiment are shown in Figure 9. From this, it can be seen that there was no significant difference in body weight between the administered and control groups after administration of **N53·HCl** and control **N53**, indicating that the dose was safe to administer. The tumor inhibition rate of **N53·HCl** at a dose of 15 mg/kg was 53.7% compared with 34.7% for the control **N53**, which was able to reduce the growth rate of tumor volume and body weight to some extent after administration compared with the blank group (Figure 9).

In conclusion, **N53·HCl** showed strong growth of subcutaneous graft against colon cancer at a dose of 15 mg/kg, and due to the improved solubility and bioavailability after salt formation, the growth of subcutaneous graft against colon cancer *in vivo* was stronger than that of **N53**, and the further development and utilization of this compound at a later stage may start from oral administration.

## 4 Conclusion

Our previous study obtained a novel anti-colon cancer compound **N53** with potential dual inhibitory activity of Topo I and COX-2. However, its poor solubility, unsatisfactory pharmacokinetic properties, and moderate *in vivo* anti-tumor activity hampered further development. This study further explored the feasibility of N53 salt formation to solve the above limitations and improve its druggability. The results showed that **N53** could only form salts with *hydrochloric acid* and *hydrobromic acid*, but not with other organic acids and inorganic acids, mainly because the relatively high pKa of these acids is conducive to the salt formation of **N53**. Intriguingly, as the water solubility of **N53·HCl** increases, the Topo1 and COX-2 inhibitory activity and anti-colon cancer activity *in vitro* are also significantly enhanced. This is mainly because the solubility of **N53** is less than 0.32 μg/mL (equivalent to 0.74 μM), which cannot be completely dissolved at the test concentration, while the solubility of **N53·HCl** is 540 μg/mL (equivalent to 1.15 mM), which is completely dissolved under the test concentration and the effective concentration increases. On the other hand, *in vivo* pharmacokinetic studies, the area under the concentration-time curve (AUC_0–t_) of **N53·HCl** at the same dose was almost three times that of **N53**, and the T1/2 extension was nearly two times. It can be seen that the salt of **N53** generates **N53·HCl**, which can greatly enhance its exposure level and prolong its elimination time in the body. Consistently, the tumor inhibition rate of **N53·HCl** *in vivo* was 53.7%, which was better than that of **N53** at 34.7% and there was no obvious toxicity. This further verified that the superior solubility increases the effective anti-tumor concentration and obtained better pharmacokinetic properties, further promoting the anti-transplanted tumor activity *in vivo*. Taken together, in view of the above-mentioned druggability advantages of the hydrochloride, **N53·HCl** is expected to be developed as a new type of colon cancer treatment drug.

## Data Availability

The datasets presented in this study can be found in online repositories. The names of the repository/repositories and accession number(s) can be found in the article/Supplementary Material.
