# Supplementary material for: Novel N-phenyl-2-(aniline) benzamide hydrochloride salt development for colon cancer therapy
Source: Front Pharmacol. 2024 Oct 30;15:1452904. doi: 10.3389/fphar.2024.1452904 (PMC11570809; doi:10.3389/fphar.2024.1452904)
Supplement: Supplementary file 1 [file DataSheet1.docx]

**Novel *N*-phenyl-2-(aniline) benzamide hydrochloride salt development for colon cancer therapy**

**Table of Contents**

^1^H NMR and ^13^C NMR for all synthesized compounds**.......................................................................S2**

HPLC results for all synthesized compounds**......................................................................................S4**

Crystal Date and Cif Check Report for **N53·HCl...............................................................................S6**

**^1^H NMR and ^13^C NMR for all synthesized compounds**

**2-((3-chloro-2-methylphenyl)amino)-N-(4-(4-methylp-iperazin-1-yl)phenyl)benzamide(N53)**

**^1^H NMR Spectrum of N53 (500 MHz, CDCL_3_)**

**^13^C NMR of N53 (126 MHz, CDCl_3_)**

**4-(4-(2-((3-chloro-2-methylphenyl)amino)ben-zamido)phenyl)-1-methylpiperazin-1-ium(N53·HCl)**

**^1^H NMR Spectrum of N53·HCl (500 MHz, DMSO-*d_6_*)**

**^^**

**^13^C NMR of N53·HCl (126 MHz, DMSO-*d_6_*)**

**^^**

**HPLC results for all synthesized compounds**

**N53·HCl（99.200%）**


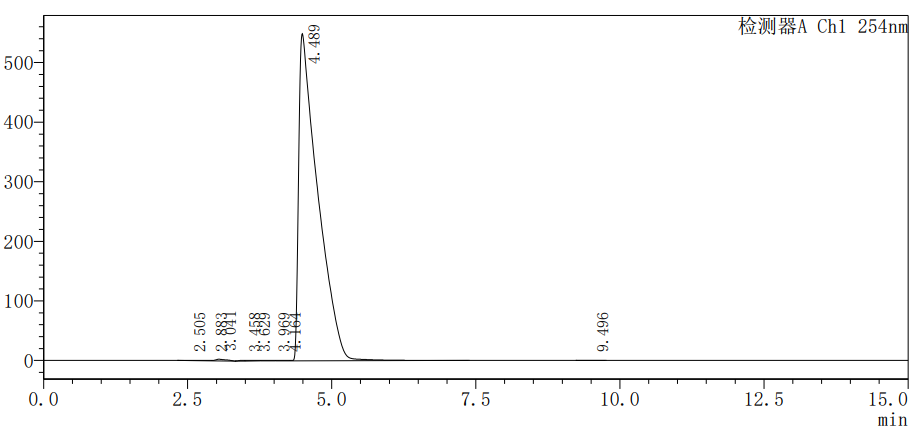


| Peak  # | RetTime  [min] | Area  [mAU*s] | Height  [mAU] | Area  % |
| --- | --- | --- | --- | --- |
| 1 | 2.505 | 2827 | 252 | 0.022 |
| 2 | 2.883 | 10539 | 741 | 0.082 |
| 3 | 3.041 | 36206 | 2804 | 0.283 |
| 4 | 3.458 | 7359 | 996 | 0.058 |
| 5 | 3.629 | 16208 | 1058 | 0.127 |
| 6 | 3.969 | 13429 | 881 | 0.105 |
| 7 | 4.164 | 13105 | 936 | 0.103 |
| 8 | 4.489 | 12675864 | 549027 | 99.200 |
| 9 | 9.496 | 2514 | 153 | 0.020 |

**N53（95.925%）**


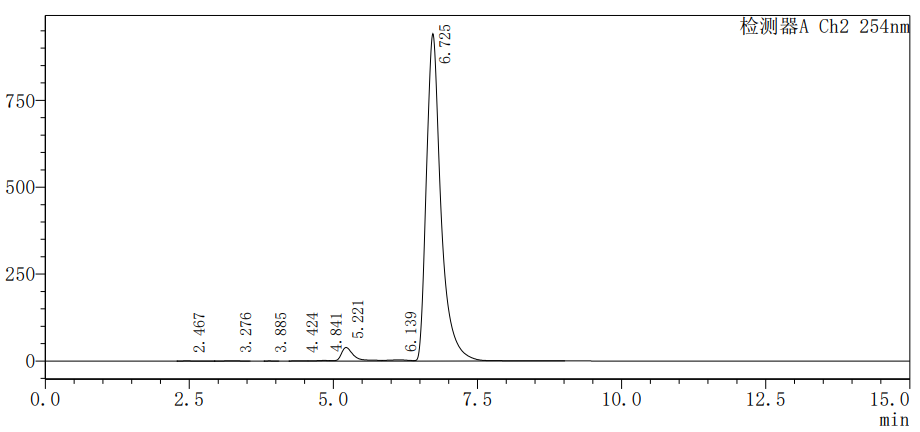


| Peak  # | RetTime  [min] | Area  [mAU*s] | Height  [mAU] | Area  % |
| --- | --- | --- | --- | --- |
| 1 | 2.467 | 2244 | 266 | 0.013 |
| 2 | 3.276 | 7747 | 762 | 0.045 |
| 3 | 3.885 | 1029 | 110 | 0.006 |
| 4 | 4.424 | 8669 | 653 | 0.050 |
| 5 | 4.841 | 22869 | 1610 | 0.132 |
| 6 | 5.221 | 582123 | 39003 | 3.366 |
| 7 | 6.139 | 79792 | 3323 | 0.461 |
| 8 | 6.725 | 16587192 | 941362 | 95.926 |

**Crystal Date and Cif Check Report for N53·HCl**

Single crystals of C_27_H_31_Cl_2_N_5_O were A suitable crystal was selected and on a 'Bruker APEX-II CCD' diffractometer. The crystal was kept at 170.0 K during data collection. Using Olex2 [1], the structure was solved with the ShelXT [2] structure solution program using Intrinsic Phasing and refined with the ShelXL [3] refinement package using Least Squares minimisation.

1. Dolomanov, O.V., Bourhis, L.J., Gildea, R.J, Howard, J.A.K. & Puschmann, H. (2009), J. Appl. Cryst. 42, 339-341.
2. Sheldrick, G.M. (2015). Acta Cryst. A71, 3-8.
3. Sheldrick, G.M. (2015). Acta Cryst. C71, 3-8.

Crystal structure determination of N53·HCl

**Crystal Data** for C_27_H_31_Cl_2_N_5_O (*M*=512.47 g/mol): monoclinic, space group P2_1_/c (no. 14), *a* = 21.9970(9) Å, *b* = 8.2672(3) Å, *c* = 14.4907(6) Å, *β* = 95.730(2)°, *V*= 2622.02(18) Å^3^, *Z* = 4, *T* = 170.0 K, μ(MoKα) = 0.277 mm^-1^, *Dcalc* = 1.298 g/cm^3^, 42212 reflections measured (5.268° ≤ 2Θ ≤ 54.228°), 5805 unique (*R*_int_ = 0.0590, R_sigma_ = 0.0351) which were used in all calculations. The final *R*_1_ was 0.0382 (I > 2σ(I)) and *wR*_2_ was 0.1029 (all data).

**(CCDC:2179821)**


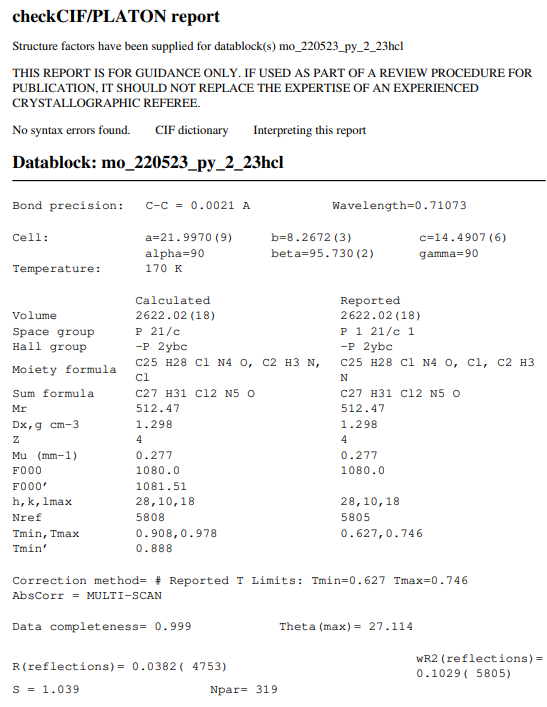


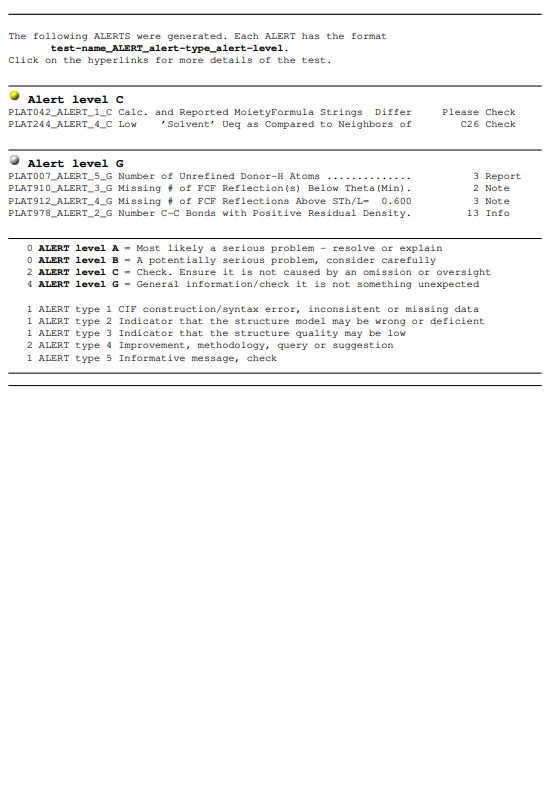


| **Table 1 Crystal data and structure refinement for N53·HCl.** | | | |  |
| --- | --- | --- | --- | --- |
| Identification code | | **N53·HCl** | |  |
| Empirical formula | | C_27_H_31_Cl_2_N_5_O | |  |
| Formula weight | | 512.47 | |  |
| Temperature/K | | 170.0 | |  |
| Crystal system | | monoclinic | |  |
| Space group | | P2_1_/c | |  |
| a/Å | | 21.9970(9) | |  |
| b/Å | | 8.2672(3) | |  |
| c/Å | | 14.4907(6) | |  |
| α/° | | 90 | |  |
| β/° | | 95.730(2) | |  |
| γ/° | | 90 | |  |
| Volume/Å^3^ | | 2622.02(18) | |  |
| Z | | 4 | |  |
| ρ_calc_g/cm^3^ | | 1.298 | |  |
| μ/mm^‑1^ | | 0.277 | |  |
| F(000) | | 1080.0 | |  |
| Crystal size/mm^3^ | | 0.43 × 0.29 × 0.08 | |  |
| Radiation | | MoKα (λ = 0.71073) | |  |
| 2Θ range for data collection/° | | 5.268 to 54.228 | |  |
| Index ranges | | -28 ≤ h ≤ 28, -10 ≤ k ≤ 9, -18 ≤ l ≤ 18 | |  |
| Reflections collected | | 42212 | |  |
| Independent reflections | | 5805 [R_int_ = 0.0590, R_sigma_ = 0.0351] | |  |
| Data/restraints/parameters | | 5805/0/319 | |  |
| Goodness-of-fit on F^2^ | | 1.039 | |  |
| Final R indexes [I>=2σ (I)] | | R_1_ = 0.0382, wR_2_ = 0.0957 | |  |
| Final R indexes [all data] | | R_1_ = 0.0507, wR_2_ = 0.1029 | |  |
| Largest diff. peak/hole / e Å^-3^ | | 0.49/-0.41 | |  |
| **Table 2 Fractional Atomic Coordinates (×10^4^) and Equivalent Isotropic Displacement Parameters (Å^2^×10^3^) for N53·HCl. U_eq_ is defined as 1/3 of of the trace of the orthogonalised U_IJ_ tensor.** | | | | |
| **Atom** | ***x*** | ***y*** | ***z*** | **U(eq)** |
| Cl1 | 5315.3(2) | 4948.2(8) | 1644.1(4) | 55.50(17) |
| O1 | 2844.0(5) | 5091.9(14) | 4285.7(9) | 32.0(3) |
| N1 | 3327.4(6) | 3314.4(17) | 2976.4(10) | 32.4(3) |
| N2 | 1872.2(5) | 4410.9(15) | 4524.9(9) | 21.7(3) |
| N3 | 1259.7(5) | 10329.9(15) | 6139.3(9) | 21.5(3) |
| N4 | 936.0(6) | 13264.0(15) | 7029.3(8) | 21.2(3) |
| C1 | 4271.4(7) | 3992(2) | 2344.8(11) | 28.1(3) |
| C2 | 4608.8(7) | 3962(2) | 1580.9(12) | 31.8(4) |
| C3 | 4414.5(8) | 3196(2) | 759.9(12) | 35.5(4) |
| C4 | 3850.0(8) | 2444(2) | 674.5(12) | 36.1(4) |
| C5 | 3491.1(7) | 2477(2) | 1404.0(12) | 32.0(4) |
| C6 | 3697.7(7) | 3231.9(19) | 2235.7(11) | 26.7(3) |
| C7 | 4492.7(9) | 4815(3) | 3239.4(13) | 44.5(5) |
| C8 | 2969.0(7) | 2087.1(18) | 3273.2(11) | 23.0(3) |
| C9 | 3050.4(7) | 479(2) | 2997.9(11) | 26.9(3) |
| C10 | 2692.5(8) | -753(2) | 3289.7(12) | 30.0(4) |
| C11 | 2240.4(8) | -432(2) | 3868.9(12) | 31.6(4) |
| C12 | 2160.1(7) | 1134.0(19) | 4164.8(11) | 25.8(3) |
| C13 | 2514.0(7) | 2415.3(18) | 3878.5(10) | 21.2(3) |
| C14 | 2430.5(7) | 4077.1(18) | 4239.2(10) | 21.6(3) |
| C15 | 1724.7(6) | 5884.5(17) | 4955.3(10) | 20.1(3) |
| C16 | 2113.7(7) | 6574.9(18) | 5658.3(10) | 22.2(3) |
| C17 | 1956.4(7) | 8010.5(18) | 6075.6(10) | 22.3(3) |
| C18 | 1405.6(6) | 8791.9(17) | 5799.0(10) | 20.0(3) |
| C19 | 1006.4(7) | 8052.4(19) | 5109.8(11) | 24.5(3) |
| C20 | 1162.6(7) | 6619.4(19) | 4699.0(11) | 24.3(3) |
| C21 | 1706.6(7) | 11144.9(18) | 6782.5(11) | 22.6(3) |
| C22 | 1569.2(7) | 12939.2(18) | 6786.1(11) | 23.7(3) |
| C23 | 482.0(7) | 12339(2) | 6401.6(11) | 27.5(3) |
| C24 | 645.3(7) | 10559(2) | 6421.7(12) | 28.0(3) |
| C25 | 800.4(8) | 15030.8(19) | 7017.1(13) | 32.6(4) |
| Cl2 | 569.9(2) | 2663.2(5) | 3904.5(3) | 27.21(11) |
| N5 | 3504.5(10) | 7741(3) | 6834.3(16) | 68.1(6) |
| C26 | 3670.4(9) | 7700(3) | 6117.8(17) | 48.2(5) |
| C27 | 3846.4(17) | 7686(4) | 5193(2) | 102.5(13) |

| **Table 3 Anisotropic Displacement Parameters (Å^2^×10^3^) for N53·HCl. The Anisotropic displacement factor exponent takes the form: -2π^2^[h^2^a*^2^U_11_+2hka*b*U_12_+…].** | | | | | | |
| --- | --- | --- | --- | --- | --- | --- |
| **Atom** | **U_11_** | **U_22_** | **U_33_** | **U_23_** | **U_13_** | **U_12_** |
| Cl1 | 34.6(3) | 79.0(4) | 55.2(3) | -2.1(3) | 15.9(2) | -24.4(2) |
| O1 | 27.3(6) | 26.9(6) | 43.8(7) | -10.7(5) | 13.8(5) | -7.8(5) |
| N1 | 31.7(7) | 27.6(7) | 41.0(8) | -11.4(6) | 18.9(6) | -7.7(6) |
| N2 | 21.1(6) | 17.8(6) | 26.8(7) | -3.4(5) | 5.4(5) | -2.2(5) |
| N3 | 17.3(6) | 22.3(6) | 25.0(6) | -5.8(5) | 2.7(5) | 0.8(5) |
| N4 | 25.5(6) | 19.2(6) | 19.3(6) | 0.2(5) | 4.6(5) | 2.4(5) |
| C1 | 25.4(8) | 28.8(8) | 30.4(8) | 1.2(7) | 5.0(6) | -1.5(6) |
| C2 | 22.8(8) | 36.1(9) | 37.6(9) | 6.3(7) | 7.6(7) | -2.7(7) |
| C3 | 31.8(9) | 45.6(11) | 30.6(9) | 3.3(8) | 11.6(7) | 0.9(8) |
| C4 | 36.1(9) | 44.1(11) | 28.5(9) | -5.0(8) | 4.1(7) | -0.2(8) |
| C5 | 23.4(8) | 36.4(9) | 36.3(9) | -5.0(7) | 3.7(7) | -3.3(7) |
| C6 | 23.4(7) | 25.3(8) | 32.6(8) | -0.2(7) | 8.8(6) | 1.1(6) |
| C7 | 42.6(11) | 54.4(12) | 37.1(10) | -7.2(9) | 7.3(8) | -17.5(9) |
| C8 | 19.3(7) | 24.8(8) | 24.8(8) | -1.0(6) | 1.9(6) | 0.3(6) |
| C9 | 25.0(8) | 27.5(8) | 29.0(8) | -3.5(7) | 5.9(6) | 4.2(6) |
| C10 | 37.2(9) | 20.7(8) | 32.8(9) | -0.9(7) | 6.7(7) | 5.3(7) |
| C11 | 41.7(10) | 20.8(8) | 34.2(9) | 3.5(7) | 13.6(7) | -1.8(7) |
| C12 | 30.5(8) | 23.2(8) | 24.9(8) | 1.2(6) | 9.0(6) | 1.3(6) |
| C13 | 21.0(7) | 20.8(7) | 21.6(7) | -1.0(6) | 1.5(6) | 1.3(6) |
| C14 | 22.8(7) | 21.9(7) | 20.3(7) | 0.3(6) | 3.6(6) | -0.4(6) |
| C15 | 22.9(7) | 17.8(7) | 20.4(7) | -0.1(6) | 6.3(6) | -1.6(5) |
| C16 | 22.2(7) | 21.0(7) | 23.4(7) | 1.5(6) | 1.9(6) | 1.9(6) |
| C17 | 22.2(7) | 22.6(7) | 21.5(7) | -1.3(6) | 0.1(6) | -1.0(6) |
| C18 | 21.7(7) | 20.1(7) | 19.0(7) | -0.7(6) | 5.2(5) | -0.8(5) |
| C19 | 19.3(7) | 26.4(8) | 27.3(8) | -4.8(6) | 0.2(6) | 2.8(6) |
| C20 | 22.4(7) | 25.6(8) | 24.6(8) | -4.9(6) | 0.0(6) | -1.4(6) |
| C21 | 20.6(7) | 21.6(7) | 25.5(8) | -3.1(6) | 0.9(6) | -0.3(6) |
| C22 | 21.8(7) | 22.9(8) | 27.1(8) | -1.5(6) | 5.0(6) | -0.8(6) |
| C23 | 21.2(7) | 34.0(9) | 27.3(8) | -7.4(7) | 1.9(6) | 4.6(6) |
| C24 | 19.3(7) | 30.2(8) | 35.3(9) | -11.1(7) | 6.7(6) | -2.3(6) |
| C25 | 39.3(9) | 21.0(8) | 39.0(10) | 2.4(7) | 12.0(8) | 8.2(7) |
| Cl2 | 24.54(19) | 34.9(2) | 22.95(19) | -4.13(15) | 6.28(14) | -4.46(15) |
| N5 | 64.4(13) | 76.9(15) | 62.9(14) | 13.2(11) | 6.4(11) | -34.6(11) |
| C26 | 38.6(11) | 45.3(12) | 59.1(14) | 7.9(10) | -3.4(10) | -19.8(9) |
| C27 | 112(3) | 127(3) | 70.8(19) | -22.5(19) | 18.6(18) | -78(2) |

| **Table 4 Bond Lengths for N53·HCl.** | | | | | | |
| --- | --- | --- | --- | --- | --- | --- |
| **Atom** | **Atom** | **Length/Å** |  | **Atom** | **Atom** | **Length/Å** |
| Cl1 | C2 | 1.7494(17) |  | C5 | C6 | 1.393(2) |
| O1 | C14 | 1.2345(18) |  | C8 | C9 | 1.405(2) |
| N1 | C6 | 1.412(2) |  | C8 | C13 | 1.421(2) |
| N1 | C8 | 1.380(2) |  | C9 | C10 | 1.379(2) |
| N2 | C14 | 1.3627(18) |  | C10 | C11 | 1.389(2) |
| N2 | C15 | 1.4208(18) |  | C11 | C12 | 1.381(2) |
| N3 | C18 | 1.4123(18) |  | C12 | C13 | 1.402(2) |
| N3 | C21 | 1.4513(19) |  | C13 | C14 | 1.488(2) |
| N3 | C24 | 1.4630(18) |  | C15 | C16 | 1.386(2) |
| N4 | C22 | 1.4949(18) |  | C15 | C20 | 1.394(2) |
| N4 | C23 | 1.493(2) |  | C16 | C17 | 1.391(2) |
| N4 | C25 | 1.4905(19) |  | C17 | C18 | 1.396(2) |
| C1 | C2 | 1.393(2) |  | C18 | C19 | 1.403(2) |
| C1 | C6 | 1.405(2) |  | C19 | C20 | 1.384(2) |
| C1 | C7 | 1.502(2) |  | C21 | C22 | 1.514(2) |
| C2 | C3 | 1.377(3) |  | C23 | C24 | 1.514(2) |
| C3 | C4 | 1.383(3) |  | N5 | C26 | 1.135(3) |
| C4 | C5 | 1.381(2) |  | C26 | C27 | 1.432(4) |

| **Table 5 Bond Angles for N53·HCl.** | | | | | | | | |
| --- | --- | --- | --- | --- | --- | --- | --- | --- |
| **Atom** | **Atom** | **Atom** | **Angle/˚** |  | **Atom** | **Atom** | **Atom** | **Angle/˚** |
| C8 | N1 | C6 | 126.31(13) |  | C9 | C10 | C11 | 120.59(15) |
| C14 | N2 | C15 | 124.01(12) |  | C12 | C11 | C10 | 119.23(15) |
| C18 | N3 | C21 | 118.63(12) |  | C11 | C12 | C13 | 121.63(14) |
| C18 | N3 | C24 | 117.57(12) |  | C8 | C13 | C14 | 120.83(13) |
| C21 | N3 | C24 | 110.23(12) |  | C12 | C13 | C8 | 118.98(13) |
| C23 | N4 | C22 | 110.28(11) |  | C12 | C13 | C14 | 120.14(13) |
| C25 | N4 | C22 | 111.30(12) |  | O1 | C14 | N2 | 121.82(14) |
| C25 | N4 | C23 | 111.95(13) |  | O1 | C14 | C13 | 122.04(13) |
| C2 | C1 | C6 | 116.52(15) |  | N2 | C14 | C13 | 116.13(13) |
| C2 | C1 | C7 | 122.80(15) |  | C16 | C15 | N2 | 121.62(13) |
| C6 | C1 | C7 | 120.67(15) |  | C16 | C15 | C20 | 118.83(14) |
| C1 | C2 | Cl1 | 119.40(13) |  | C20 | C15 | N2 | 119.48(13) |
| C3 | C2 | Cl1 | 117.18(13) |  | C15 | C16 | C17 | 120.57(14) |
| C3 | C2 | C1 | 123.41(15) |  | C16 | C17 | C18 | 121.08(14) |
| C2 | C3 | C4 | 118.83(16) |  | C17 | C18 | N3 | 122.50(13) |
| C5 | C4 | C3 | 119.95(17) |  | C17 | C18 | C19 | 117.81(13) |
| C4 | C5 | C6 | 120.60(15) |  | C19 | C18 | N3 | 119.54(13) |
| C1 | C6 | N1 | 118.21(15) |  | C20 | C19 | C18 | 120.96(14) |
| C5 | C6 | N1 | 121.08(14) |  | C19 | C20 | C15 | 120.66(14) |
| C5 | C6 | C1 | 120.65(15) |  | N3 | C21 | C22 | 109.54(12) |
| N1 | C8 | C9 | 120.96(14) |  | N4 | C22 | C21 | 111.59(12) |
| N1 | C8 | C13 | 120.76(14) |  | N4 | C23 | C24 | 110.06(12) |
| C9 | C8 | C13 | 118.27(14) |  | N3 | C24 | C23 | 110.15(13) |
| C10 | C9 | C8 | 121.26(14) |  | N5 | C26 | C27 | 176.7(3) |

| **Table 6 Torsion Angles for N53·HCl.** | | | | | | | | | | |
| --- | --- | --- | --- | --- | --- | --- | --- | --- | --- | --- |
| **A** | **B** | **C** | **D** | **Angle/˚** |  | **A** | **B** | **C** | **D** | **Angle/˚** |
| Cl1 | C2 | C3 | C4 | -178.11(14) |  | C9 | C10 | C11 | C12 | -1.2(3) |
| N1 | C8 | C9 | C10 | -179.80(15) |  | C10 | C11 | C12 | C13 | 1.5(3) |
| N1 | C8 | C13 | C12 | -179.92(14) |  | C11 | C12 | C13 | C8 | -0.4(2) |
| N1 | C8 | C13 | C14 | -2.6(2) |  | C11 | C12 | C13 | C14 | -177.72(15) |
| N2 | C15 | C16 | C17 | -179.35(13) |  | C12 | C13 | C14 | O1 | 154.05(15) |
| N2 | C15 | C20 | C19 | 179.76(14) |  | C12 | C13 | C14 | N2 | -25.3(2) |
| N3 | C18 | C19 | C20 | 173.82(14) |  | C13 | C8 | C9 | C10 | 1.2(2) |
| N3 | C21 | C22 | N4 | 57.13(16) |  | C14 | N2 | C15 | C16 | -45.4(2) |
| N4 | C23 | C24 | N3 | -58.42(17) |  | C14 | N2 | C15 | C20 | 137.74(15) |
| C1 | C2 | C3 | C4 | 1.4(3) |  | C15 | N2 | C14 | O1 | -4.5(2) |
| C2 | C1 | C6 | N1 | -176.55(15) |  | C15 | N2 | C14 | C13 | 174.85(13) |
| C2 | C1 | C6 | C5 | 0.8(2) |  | C15 | C16 | C17 | C18 | 0.0(2) |
| C2 | C3 | C4 | C5 | 0.3(3) |  | C16 | C15 | C20 | C19 | 2.8(2) |
| C3 | C4 | C5 | C6 | -1.4(3) |  | C16 | C17 | C18 | N3 | -173.34(14) |
| C4 | C5 | C6 | N1 | 178.11(16) |  | C16 | C17 | C18 | C19 | 2.2(2) |
| C4 | C5 | C6 | C1 | 0.8(3) |  | C17 | C18 | C19 | C20 | -1.9(2) |
| C6 | N1 | C8 | C9 | 15.5(3) |  | C18 | N3 | C21 | C22 | 159.64(12) |
| C6 | N1 | C8 | C13 | -165.52(15) |  | C18 | N3 | C24 | C23 | -157.79(13) |
| C6 | C1 | C2 | Cl1 | 177.56(13) |  | C18 | C19 | C20 | C15 | -0.6(2) |
| C6 | C1 | C2 | C3 | -2.0(3) |  | C20 | C15 | C16 | C17 | -2.5(2) |
| C7 | C1 | C2 | Cl1 | -1.2(2) |  | C21 | N3 | C18 | C17 | 1.6(2) |
| C7 | C1 | C2 | C3 | 179.26(18) |  | C21 | N3 | C18 | C19 | -173.85(14) |
| C7 | C1 | C6 | N1 | 2.3(2) |  | C21 | N3 | C24 | C23 | 61.99(17) |
| C7 | C1 | C6 | C5 | 179.62(17) |  | C22 | N4 | C23 | C24 | 54.33(16) |
| C8 | N1 | C6 | C1 | -140.64(17) |  | C23 | N4 | C22 | C21 | -54.23(16) |
| C8 | N1 | C6 | C5 | 42.0(3) |  | C24 | N3 | C18 | C17 | -135.19(15) |
| C8 | C9 | C10 | C11 | -0.1(3) |  | C24 | N3 | C18 | C19 | 49.31(19) |
| C8 | C13 | C14 | O1 | -23.2(2) |  | C24 | N3 | C21 | C22 | -60.62(16) |
| C8 | C13 | C14 | N2 | 157.44(14) |  | C25 | N4 | C22 | C21 | -179.11(13) |
| C9 | C8 | C13 | C12 | -0.9(2) |  | C25 | N4 | C23 | C24 | 178.84(13) |
| C9 | C8 | C13 | C14 | 176.37(14) |  |  |  |  |  |  |

| **Table 7 Hydrogen Atom Coordinates (Å×10^4^) and Isotropic Displacement Parameters (Å^2^×10^3^) for N53·HCl.** | | | | |
| --- | --- | --- | --- | --- |
| **Atom** | ***x*** | ***y*** | ***z*** | **U(eq)** |
| H1 | 3325.05 | 4238.1 | 3277.85 | 39 |
| H2 | 1585.59 | 3668.47 | 4437.52 | 26 |
| H4 | 909.64 | 12865.11 | 7675.63 | 25 |
| H3 | 4663.81 | 3185.46 | 261.06 | 43 |
| H4A | 3709.29 | 1905.49 | 114.51 | 43 |
| H5 | 3099.79 | 1979.9 | 1337.68 | 38 |
| H7A | 4298.46 | 5879.06 | 3264.22 | 67 |
| H7B | 4937.16 | 4948.64 | 3275.97 | 67 |
| H7C | 4386.98 | 4155.27 | 3762 | 67 |
| H9 | 3357.9 | 235.47 | 2603.1 | 32 |
| H10 | 2755.71 | -1830.66 | 3092.85 | 36 |
| H11 | 1989.55 | -1279.89 | 4059.03 | 38 |
| H12 | 1857.37 | 1348.34 | 4572.61 | 31 |
| H16 | 2490.9 | 6063.37 | 5856.79 | 27 |
| H17 | 2228.29 | 8466.55 | 6556.66 | 27 |
| H19 | 623.18 | 8541.94 | 4922.06 | 29 |
| H20 | 884.1 | 6131.93 | 4237.9 | 29 |
| H21A | 2121.87 | 10964.99 | 6594.61 | 27 |
| H21B | 1692.55 | 10697.52 | 7413.69 | 27 |
| H22A | 1866.66 | 13485.81 | 7240.6 | 28 |
| H22B | 1616.43 | 13394.86 | 6165.46 | 28 |
| H23A | 478.08 | 12756.12 | 5760.55 | 33 |
| H23B | 68.53 | 12485.69 | 6602.89 | 33 |
| H24A | 627.74 | 10128.88 | 7056.1 | 34 |
| H24B | 346.15 | 9955.91 | 5996.75 | 34 |
| H25A | 1086.27 | 15581.74 | 7475.33 | 49 |
| H25B | 381.24 | 15205.41 | 7169.52 | 49 |
| H25C | 844.55 | 15466.77 | 6398.47 | 49 |
| H27A | 3623.11 | 6829.6 | 4835.09 | 154 |
| H27B | 4286.47 | 7483.72 | 5212.24 | 154 |
| H27C | 3750.04 | 8734.4 | 4898.44 | 154 |
